# Protozoan Parasites of Rodents and Their Zoonotic Significance in Boyer-Ahmad District, Southwestern Iran

**DOI:** 10.1155/2016/3263868

**Published:** 2016-02-21

**Authors:** Zeinab Seifollahi, Bahador Sarkari, Mohammad Hossein Motazedian, Qasem Asgari, Mohammad Javad Ranjbar, Samaneh Abdolahi Khabisi

**Affiliations:** Department of Parasitology and Mycology, School of Medicine, Shiraz University of Medical Sciences, Shiraz, Iran

## Abstract

*Backgrounds*. Wild rodents are reservoirs of various zoonotic diseases, such as toxoplasmosis, babesiosis, and leishmaniasis. The current study aimed to assess the protozoan infection of rodents in Boyer-Ahmad district, southwestern Iran.* Materials and Methods*. A total of 52 rodents were collected from different parts of Boyer-Ahmad district, in Kohgiluyeh and Boyer-Ahmad province, using Sherman live traps. Each rodent was anesthetized with ether, according to the ethics of working with animals, and was dissected. Samples were taken from various tissues and stool samples were collected from the contents of the colon and small intestines. Moreover, 2 to 5 mL of blood was taken from each of the rodents and the sera were examined for anti-*Leishmania* antibodies, by ELISA, or anti-*T. gondii* antibodies, by modified agglutination test (MAT). DNA was extracted from brain tissue samples of each rodent and PCR was used to identify the DNA of* T. gondii*.* Results*. Of the 52 stool samples of rodents studied by parasitological methods, intestinal protozoa infection was seen in 28 cases (53.8%). From 52 rodents, 19 (36.5%) were infected with* Trichomonas*, 10 (19.2%) with* Giardia muris*, and 11 (21.2%) with* Entamoeba *spp. Also, 10 cases (19.2%) were infected with* Blastocystis*, 3 (5.8%) were infected with* Chilomastix*, 7 (13.5%) were infected with* Endolimax*, 1 (1.9%) was infected with* Retortamonas*, 3 (5.77%) were infected with* T. gondii*, and 6 (11.54%) were infected with* Trypanosoma lewisi*. Antibodies to* T. gondii* were detected in the sera of 5 (9.61%) cases. Results of the molecular study showed* T. gondii* infection in 3 (5.77%) of the rodents. Findings of this study showed that rodents in Kohgiluyeh and Boyer-Ahmad province, southwestern Iran, are infected with several blood and intestinal parasites; some of them might be potential risks to residents and domestic animals in the region.

## 1. Introduction

Rodents are the most frequent and important mammals on the Earth, because they can adapt themselves to the different locations and environmental changes. These animals live on almost every continent except Antarctica [1]. Rodents are considered as reservoirs of various zoonotic diseases including toxoplasmosis, babesiosis, and leishmaniasis [1–4]. Nevertheless, rodents cannot directly cause disease in humans and disease is mainly transmitted to humans if human is in contact with rodents' feces and secretory materials. Transmission of the zoonotic pathogens to humans can occur via rodent's urine, feces, hair, and saliva [2]. Human activities which change the ecosystem of rodents' living place have an important role in the epidemiology of zoonotic diseases. Given the damage of rodents to humans and economic loss and due to their health importance, parasitological studies on rodents seem necessary [2]. Several studies have been done on parasitic infections of wild rodent in Iran [5–10]. However, due to ecological differences in different areas of the country, the parasitic fauna of the rodents in each ecological setting might be different. This notion justifies new studies on parasitic infection of the rodents in other areas of the country. The current study aimed to assess the parasitic protozoan infections of rodents in Boyer-Ahmad district, southwest Iran.

## 2. Materials and Methods

### 2.1. The Study Area

Boyer-Ahmad district is located in Kohgiluyeh and Boyer-Ahmad province. The province is located in southwest of Iran with geographical coordinates of 30° 40′ 12^″^ N, 51° 36′ 0^″^ E. The province has two types of tropical and cold climate and Boyer-Ahmad district is located in the cold area. The mean of long-term rain and snow amount is above 600 mm in this area and a wide area of the county is covered with forests of oak, wild pistachio, and mountain almond. The main professions of the people are agricultural practice and breeding and raising livestock.

### 2.2. Rodents' Collection and Identification

Considering the map of the study area, 52 rodents were collected from different parts of Boyer-Ahmad County, using Sherman live traps with roasted almonds, as bait, in the summer and autumn of 2014. Different areas of the district, including villages of Kakan, Madvan, Tange Sorkh, Kal Morgah, Mansourabad, and Dehno, were selected for sampling. After transferring to the laboratory, the genus and species of rodents were identified based on morphological characteristics. This was done to subsequently find out the rate of protozoan parasites in each rodent's species.

### 2.3. Evaluation of Rodents' Protozoan Infection

After transferring the rodents to the laboratory, they were anesthetized with ether and blood samples were taken from their heart. Different rodent parts were carefully examined and necessary samples were prepared. Smears were prepared from rodent liver, spleen, and peripheral blood on glass slides, fixed with methanol, and stained with Giemsa. Then smears were studied by an optical microscope with 100x magnification.

Temporary staining of rodent's stool samples, with Lugol's solution, was done for detection of any protozoan cysts or trophozoites. The samples were also examined with formalin-ethyl acetate sedimentation and zinc sulfate floatation techniques and the obtained materials were observed by conventional light microscope. Smears were also prepared from the rodent stool samples and stained with trichrome. Moreover, smears were prepared from the stool sediments or floated materials, obtained by concentration methods, fixed with methanol, and stained with acid-fast staining to detect coccidia parasites in fecal samples.

### 2.4. Serological Assessment of Rodents' Sera Samples

Rodents' sera were examined by indirect ELISA for anti-*Leishmania* antibodies. Moreover, MAT was performed on rodent sera samples, as previously described, to assess anti-*T. gondii* antibodies [11]. Sera were studied in two dilutions of 1 : 20 and 1 : 40 and samples with MAT titer of 1 : 40 or higher were considered as positive.

### 2.5. Molecular Analysis of Rodents' Tissue Samples

DNA was extracted from brain tissue samples of each rodent, using DNA extraction kit, based on the manufacturer's (Yekta-Tajhiz Azma, Tehran, Iran) instructions. PCR was performed to amplify a 529 bp gene of* T. gondii*, as described by Edvinsson et al. [12]. The two primers used were TOXOF CAG GGA GGA AGA CGA AAGTTG and TOXOR CAG ACA CAG TGC ATC TGG ATT. PCR products were separated in 1.5% agarose gel and stained with ethidium bromide.

### 2.6. Statistical Analysis

The statistical analysis was performed with SPSS software (version 16). Chi-square test was used to examine the association between rodent's parasitic infections and related studied factors, such as rodent's species, gender, place of collection, and weight.

## 3. Results

A total of 52 rodents were captured during the course of this study, including 25 (48.1%)* Meriones*, 15 (28.8%)* Rattus*, 10 (19.2%)* Apodemus*, 1 (1.9%)* Calomyscus*, and 1 (1.9%)* Arvicola*. Among the captured rodents, 28 (53.8%) were males and 24 (46.2%) were females.

**Figure 1:**
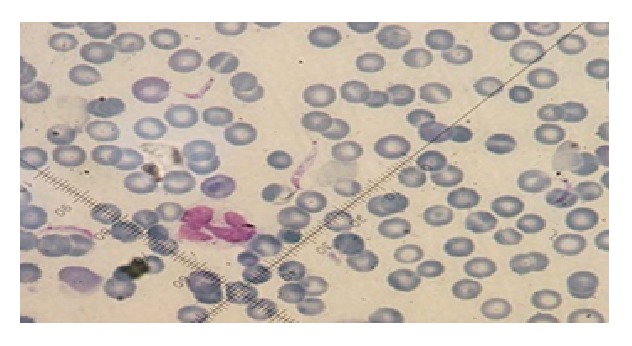
*Trypanosoma lewisi* in blood smear of the studied rodents, stained with Giemsa (100x).

**Figure 2:**
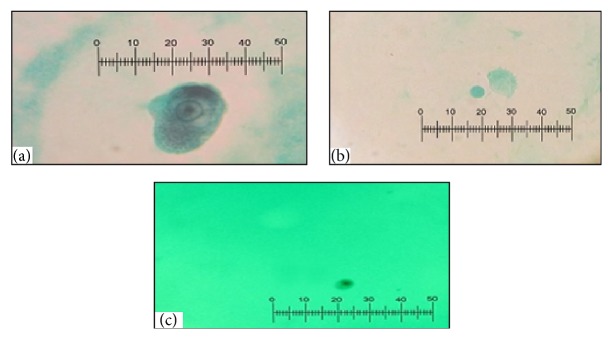
Protozoa in stool samples of the rodents, stained with trichrome. (a)* Entamoeba* trophozoite, (b)* Trichomonas* trophozoite, and (c)* Endolimax* trophozoite (100x).

Of the 52 feces samples of rodents, examined by parasitological methods, 37 (71.1%) were infected with at least one protozoan parasite, whereas 15 (28.8%) of the rodents were not infected with any intestinal protozoan parasites. From 52 rodents, 19 (36.5%) were infected with* Trichomonas*, 10 (19.2%) with* G. muris*, 11 (21.2%) with* Entamoeba*, 10 (19.2%) with* Blastocystis*, 3 (5.8%) with* Chilomastix*, 7 (13.5%) with* Endolimax*, and 1 (1.9%) was infected with* Retortamonas*. Regarding the rodents' infection with blood and tissue protozoa, 3 (5.77%) were infected with* T. gondii* and 6 (11.54%) with* Trypanosoma lewisi* (Figure 1). Anti-*Leishmania* antibodies were detected in the sera of 8 (15.34%) of the rodents; among them were 6 rodents which were also infected with* Trypanosoma lewisi*. No* Leishmania* parasites were observed in the impression smears of liver or spleen of the seropositive rodents.  Figure 2 shows a few of intestinal protozoa detected in trichrome-stained samples of rodents' feces.

Multiple infections were seen in 19 out of 52 (36.5%) rodents. Simultaneous infection with* Trichomonas* and* Entamoeba* was seen in 5.8%,* Trichomonas* and* G. muris* in 1.9%,* G. muris* and* Trypanosoma* in 1.9%,* Blastocystis* and* G. muris* in 1.9%,* Trichomonas* and* Blastocystis* in 1.9%, and* Entamoeba* and* Blastocystis* in 1.9% of the rodents. Also, simultaneous infection with* Blastocystis*,* Trypanosoma*, and* Endolimax* was observed in 1.9% of the rodents.

In this study, 54.2% of rodents, infected with intestinal protozoa, were female and 45.8% were male. Statistical analysis showed no significant correlation between various protozoa and gender of the rodents (*P* > 0.05).

The highest rates of infection with* G. muris* (70%),* Trichomonas* (36.8%),* Endolimax* (71.4%),* Trypanosoma* (100%), and* Blastocystis* (60%) were seen in* Rattus* genus and the highest infection with* Entamoeba* (54.5%) was seen in the genus* Meriones*. Statistical analysis showed significant correlation between protozoa infection and the rodent's genus (*P* < 0.05).  Table 1 shows the distribution of protozoan infections according to the rodent genus.

**Figure 3:**
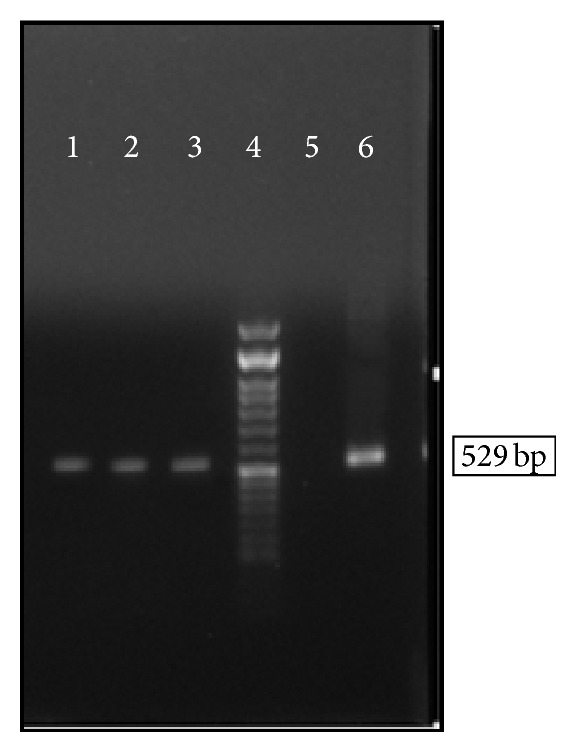
The PCR product of the DNA of* T. gondii* isolated from the rodents brain tissues. Lanes 1–3, samples isolated from rodents' brain tissue; lane 4, 50 bp DNA Ladder; lane 5, negative control; lane 6, positive control (tachyzoite prepared from mice peritonea).

Findings of the molecular study showed* T. gondii* infection in 3 (5.77%) rodents; two male and one female. Rodents infected with* T. gondii* were from* Apodemus*,* Meriones*, and* Calomyscus* genus.  Figure 3 shows PCR products of DNA, isolated from rodents' brain tissue.

No cases of coccidial infection were seen in any of the fecal samples when the samples were evaluated by modified acid-fast staining method.

## 4. Discussion

Rodents are considered as reservoirs for a few of helminthic and protozoan parasites [1, 2]. Among the protozoa parasites of rodents is* T. gondii* which is common in rodents and these animals can behave as natural reservoir for this protozoa. Evaluation of* T. gondii* infection in rodents, as the main pray for cat, with regards to the role of cat in spreading of* T. gondii* oocyst in the environment, is important [13]. Rate of* Toxoplasma* infection in rodents is different based on ecological status of a given area. In the current study,* T. gondii* infection was common protozoa of the studied rodents. Saki and Khademvatan reported a prevalence rate of 6% for* T. gondii* in rodents of Ahvaz district, south of Iran [14]. Study of Mercier et al. in 2013, assessing 766 rodents in Niamey district of Niger, revealed* Toxoplasma* infection in 1.96% of the studied rodents [15]. In the current study,* T. gondii* infection was found in* Apodemus*,* Meriones*, and* Calomyscus* genus and, to the best of our knowledge, this is the first report of molecular detection of* T. gondii* infection in* Calomyscus* from Iran.

Another important parasitic infection that rodents have an important role in its transmission, as reservoirs, is leishmaniasis. A large number of species of rodents have been identified as reservoir of cutaneous leishmaniasis in Iran [3–5]. Mohebali et al. reported the infection of different species of the rodents, including* Rhombomys opimus*,* Meriones libycus, Tatera indica*, and* Meriones hurrianae* with* L. major* [16]. Rassi et al. reported that* Meriones libycus* is the main reservoir of cutaneous leishmaniasis in Fars province, southern Iran [17]. In the present study,* Leishmania* infection was not detected in any of the studied rodents. The reason for this is that leishmaniasis is mainly seen in tropical and subtropical areas of Iran, while Boyer-Ahmad district is located in cold and mountainous region of the country and is not considered as an endemic focus of cutaneous leishmaniasis. Although visceral leishmaniasis is not uncommon in this district, its reservoirs are dogs, carnivores, or properly cats, rather than rodents [18–20].

Rodents are frequently infected with intestinal protozoa and may act as reservoir for a few of them. In the current study, intestinal protozoa including* Trichomonas*,* Entamoeba*, and* G. muris* were detected in the studied rodents. Rate of* Giardia* infection, as the main intestinal protozoa, in rodents was found to be 14.6% in Al Hindi and Abu-Haddaf study in 2013 in Palestine, 96.3% in* Microtus* and 48.3% in* Apodemus* species in Poland, 2.5% in the study by Rasti and colleagues in 2000 in Kashan, Iran, and 2.7% in the study by Kia and colleagues in 2001 in Ahvaz, south of Iran [7, 21–23]. In the current study, rate of* G. muris* infection in the rodents was relatively high. Further study is needed to compare the genotype of these protozoa, isolated from the rodents with the human isolates.

Infection with blood protozoa,* Trypanosoma,* was common in the studied rodents in our study. Lower rate of infection (10%) with this parasite has been reported in Kia et al. study in Ahvaz, south of Iran [7], whereas higher rates of infection have been reported from Brazil (21.7%) and India (82.3%) [24, 25].

In this study, infection with intestinal coccidia was not found in the rodents, while other studies have reported coccidia infection in these animals [21]. This difference could be due to the differences in climatic conditions in the studied areas, and also nutritional or habitat preferences of the rodents.

Taken together, findings of the current study revealed that rodents in Kohgiluyeh and Boyer-Ahmad province, in southwest of Iran, are infected with many intestinal and blood protozoa. Some of these protozoa may be potential risks to the residents and domestic animals in the region. High prevalence of intestinal protozoan infections in the rodents might be linked to the unsafe disposal of human waste and also use of human and animal fertilizers in the area.

## Figures and Tables

**Table 1:** Distribution of protozoan infection according to genus of the studied rodents.

	*Rattus*		*Meriones*		*Calomyscus*		*Apodemus*		*Arvicola*		Total	
	Number	%	Number	%	Number	%	Number	%	Number	%	Number	%
*G. muris*	7	70	2	20	0	0	1	10	0	0	10	19.23
*Trichomonas*	7	36.8	4	21.1	1	5.3	7	36.8	0	0	19	36.5
*Blastocystis*	6	60	2	20	1	10	1	10	0	0	10	19.23
*Entamoeba*	4	36.4	6	54.5	0	0	1	1.9	0	0	11	21.1
*Chilomastix*	1	33.3	1	33.3	0	0	1	33.3	0	0	3	5.8
*Endolimax*	5	71.4	1	14.3	0	0	1	33.3	0	0	7	13.46
*Retortamonas*	0	0	0	0	0	0	1	100	0	0	1	1.92
*Trypanosoma*	6	100	0	0	0	0	0	0	0	0	6	11.53
*T. gondii*	0	0	1	33.3	1	33.3	1	33.3	0	0	3	508
